# Preparation of Felodipine–PEG Solid Dispersions by Solvent-Free scCO_2_ Processing and Their Translation into Orally Disintegrating Tablets

**DOI:** 10.1021/acsomega.5c13416

**Published:** 2026-02-12

**Authors:** Siva S. Kolipaka, Laura A. Junqueira, Elizabeth J. Pathrapankal, Dennis Douroumis, Vivek Trivedi

**Affiliations:** † Centre for Research Innovation, University of Greenwich, Medway Campus, Chatham Maritime, Chatham ME4 4TB, U.K.; ‡ Delta Pharmaceutics Ltd., 1- 3 Manor Road, Chatham, Kent ME4 6AE, U.K.; § School of Life and Health Sciences, Department of Health Sciences, Pharmacy, University of Nicosia, 29th Street, No. 17, Elliniko, 167 77 Athens, Greece; ∥ Medway School of Pharmacy, 2240 University of Kent, Central Avenue, Chatham Maritime ME4 4TB, U.K.

## Abstract

Poor aqueous solubility remains a major limitation for the oral bioavailability of molecules such as felodipine (FDP), necessitating formulation strategies that enhance drug dissolution while remaining compatible with scalable, solvent-free processing. In this study, solid dispersions (SDs) of FDP were prepared using an organic solvent-free supercritical carbon dioxide (scCO_2_) process with four grades of polyethylene glycol (PEG 4K, 6K, 10K, and 20K) at drug loadings of 10, 20, and 30% w/w. The influence of PEG molecular weight, drug loading, and scCO_2_ processing conditions on the solid-state properties and dissolution behavior of FDP was investigated. X-ray diffraction (XRD) and differential scanning calorimetry (DSC) revealed a substantial reduction in FDP crystallinity, indicative of partial or extensive amorphization, dependent on polymer grade and processing temperature. All SDs showed markedly enhanced dissolution compared with crystalline FDP and corresponding physical mixtures, with PEG 4K SDs processed at 45 °C and PEG 20K SDs processed at 60 °C exhibiting the most pronounced improvements. Optimized SDs were subsequently incorporated into orally disintegrating tablets (ODTs), which retained the enhanced dissolution performance of the parent SDs, demonstrating that tabletting did not compromise drug release. While both PEG 4K- and PEG 20K-based ODTs showed rapid dissolution, PEG 20K formulations exhibited superior mechanical integrity, identifying 30% w/w drug-loaded PEG 20K SDs as the most suitable system for ODT development. Overall, this study demonstrates a green, solvent-free scCO_2_-based strategy for producing high-performance solid dispersions and their successful translation into ODTs for poorly water-soluble drugs.

## Graphical abstract

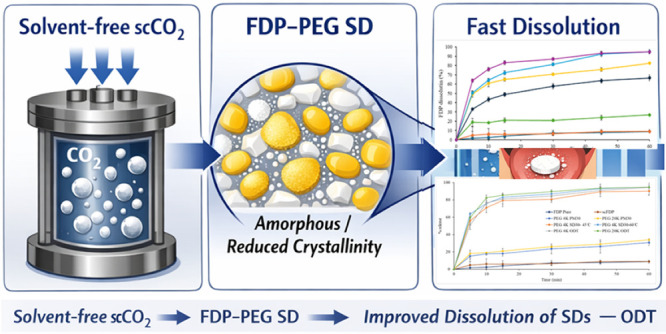

## 1 Introduction

At present, one of the major issues limiting the applications of many active pharmaceutical ingredients (APIs) is undoubtedly related to their low aqueous solubility.^1^ It is estimated that up to 70% of APIs and new chemical entities (NCEs) exhibit poor water solubility, which can lead to reduced drug bioavailability. SDs are one of the approaches to improve drug dissolution.^2,3^ SDs contain at least two distinct components: a hydrophilic matrix and a hydrophobic drug.^4^ The interaction between the drug and the polymer results in a reduction of drug crystallinity and an improvement in wettability, leading to enhanced solubility/dissolution rates of poorly soluble APIs.^5^ Despite their effectiveness, conventional strategies for improving drug dissolution are often associated with significant limitations. Different methods are available to improve the dissolution characteristics of poorly water-soluble drugs, including particle size reduction, salt formation, cocrystallization, and the use of surfactants and cosolvents, etc.^6,7^ However, these technologies have their disadvantages as they may require high temperatures and/or the use of organic solvents, neutral and weakly acidic/basic drugs have difficulty forming salts, while the use of surfactants/cosolvents can compromise the commercial viability of liquid formulations. Particle size reduction methods are highly energy-intensive and may result in the formation of fine powders with low wettability and a high tendency to form agglomerates.^8^

In light of these limitations, alternative and more sustainable processing approaches are required. Therefore, it is essential to investigate green technologies, such as supercritical fluid (SCF) processing, as an alternative for developing pharmaceutical products.^9^ An SCF is a substance above its critical pressure and temperature that has characteristics intermediate between liquids and gases, including density, viscosity, and mass transfer properties similar to gases.^10^ There are many SCFs, but scCO_2_ is more suitable for the processing of organic compounds due to its lower critical temperature (31.1 °C) and pressure (73.8 bar).^11^ CO_2_ is also nontoxic, generally recognized as safe (GRAS), inert, and inexpensive compared to many of the organic solvents available as SCF.^12^ These properties make scCO_2_ particularly attractive for the development of pharmaceutical formulations. Furthermore, the adjustable properties of scCO_2_ make it very versatile in pharmaceutical processing, as it can be used as an antisolvent, extractant, solvent, and/or plasticizer for a variety of amorphous or crystalline drugs and polymers.^13^ In addition, the easy separation of CO_2_ from the polymer matrix at the end of the formulation process ensures that only solvent-free products are produced.^14^

Enhancing drug dissolution is particularly important for dosage forms designed to deliver a rapid onset and improved patient acceptability. The United States Pharmacopoeia (USP) defines ODTs as solid oral dosage forms that swiftly disintegrate in the mouth upon contact with saliva, without mastication or liquid, and are designed for consumption without swallowing whole.^15^ Disintegration time is a vital parameter in characterization methods, which needs to be within the mandated 30 s by the US Food and Drug Administration (FDA) or 3 min according to the European Pharmacopoeia. Individuals across all age groups, including pediatrics, the elderly, and those with swallowing difficulties, and in circumstances with a need for rapid onset of action, can benefit from ODTs. They are user-friendly because of the swift absorption as ODTs disintegrate quickly, allowing the drug to be swallowed, with paragastric absorption possible for suitable drugs in the absence of water.^16^ Moreover, ODTs can be preferred over oral ingestion, given the minimal risk of choking or asphyxiation for pediatric or geriatric patients.^17,18^

The selection of an appropriate drug candidate and polymer carrier is vital for the successful development of ASD-based ODT formulations. Felodipine (FDP) was selected as a model drug in this work over frequently used BCS class II drugs like ibuprofen (IBU), because IBU is highly soluble in scCO_2_, which could restrict its interaction with PEG due to the preferred drug-scCO_2_ interaction.^19^ Moreover, IBU also poses dosing-related challenges; for example, the lowest IBU dose is ∼100 mg/tablet, which will be difficult to incorporate into ODT formulations. In contrast, FDP is a highly suitable drug candidate for this work, with its limited solubility in scCO_2_ and a maximum marketed dose of 10 mg/tablet.

Figure 1A presents the structure of FDP [4-(2,3-dichlorophenyl)-1,4-dihydro-2,6-dimethyl-3,5-pyridine carboxylic acid ethyl methyl ester] that is supplied as a light yellow, crystalline powder with a molecular weight of 384.26 g/mol. It is an inhibitor of L-type calcium channels and preferentially inhibits L-type calcium channels.^20^ It decreases arterial blood pressure and total peripheral resistance. FDP is almost insoluble in water but is soluble in various organic solvents like ethanol, DMSO (dimethyl sulfoxide), and DMF (dimethylformamide), and is sparingly soluble in aqueous buffers. For maximum solubility in aqueous buffers, FDP should be first dissolved in DMSO and then diluted with the aqueous buffer with a solubility of approximately 0.25 mg/mL in a 1:3 solution of DMSO: PBS (pH 7.2).^21^

**Figure 1:**
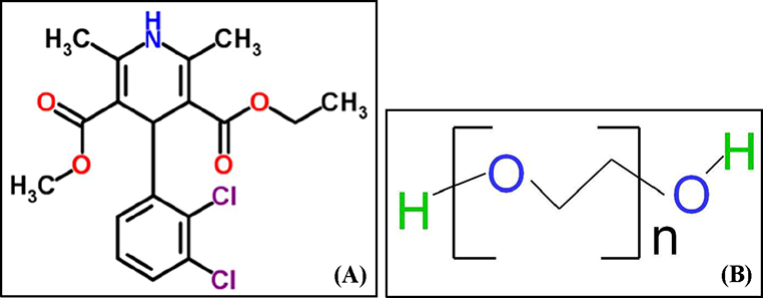
Structure of (A) FDP and (B) polyethylene glycol (*n*, number of ethylene glycol units).

Alongside the choice of API, polymer selection plays a key role in ASD performance. Figure 1B presents the structure of polyethylene glycol (PEG), which is commonly expressed as H–(O–CH_2_–CH_2_)_*n*_–OH. PEG is a synthetic, hydrophilic, biocompatible polymer that is FDA-approved, nontoxic, and nonimmunogenic; hence, it is widely employed in biomedical and various pharmaceutical applications.^22^ Ring-opening polymerization of ethylene oxide produces PEGs with a broad range of molecular weights. PEGs can be synthesized as linear, branched, Y-shaped, or multiarm polymers. The terminal hydroxyl end group of PEGs may be replaced with reactive functional end groups to facilitate cross-linking and conjugation chemistries.

PEGs are used in bioconjugation, drug delivery, surface functionalization, and tissue engineering. Three-dimensional, water-swollen PEG hydrogels are known to resist protein attachment and biodegradation. Cross-linking reactive PEG end groups creates PEG hydrogels, which are used in tissue engineering and medication delivery.^23^ PEG can improve the dissolution rate of poorly soluble drugs through the formation of micelles and as a polymer matrix in SDs.^24^ The nonionic and hydrophilic properties of PEG, along with its surface-active characteristics, can help in sustaining the solubility of a loaded hydrophobic drug while maintaining the drug supersaturation throughout the gastrointestinal tract. PEG has been proven to enhance the dissolution rate of BCS class II drugs via the formation of ASDs.^24^

There are a few reports that describe the development of SDs using PEG via scCO_2_ processing, alone or in combination with other additives, such as carbamazepine SDs with PEG 4K and TPGS, and atorvastatin SDs with PEG 6K.^25,26^ However, to the best of our knowledge, no studies have reported the development of SDs using PEG as the sole carrier matrix for FDP via scCO_2_ processing, with a specific focus on how the polymer’s molecular weight influences drug amorphization and dissolution. Hence, this work aims to study the potential of scCO_2_ as a processing medium to prepare SD of FDP in PEG at low temperatures and pressures without the use of organic solvents, thereby offering a more sustainable and environmentally benign approach to enhance the dissolution profile of poorly water-soluble drug and their translation into ODTs.

## 2 Materials and Methods

FDP and PEG (4K, 6K, 10K, and 20K Da) were supplied by Merck KGaA (Darmstadt, Germany). Liquid CO_2_ (99.9%) was supplied by BOC Ltd., Guildford, UK. Microcrystalline Avicel (MCC) 102 was supplied by IFF Pharma Solution (Newark, USA). Sodium stearyl fumarate and croscarmellose sodium were supplied by JRS Pharma (Rosenberg, Germany). Orange flavor and Sucralose were obtained from Merck (Darmstadt, Germany). Other chemicals used in dissolution studies were deionized water (University of Greenwich, UK), monobasic sodium phosphate, and dibasic sodium phosphate anhydrous, sodium hydroxide, hydrochloric acid, and sodium lauryl sulfate, which were obtained from Sigma-Aldrich (Buchs, Switzerland).

### 2.1 Solid–Liquid (S-L) Transition of Polymer in scCO_2_

The S-L transition parameters for all PEG 4K, 6K, 10K, and 20K and the corresponding physical mixtures (PMs) containing 10% FDP were determined using a Phase Monitor (SFT Phase Monitor II, Supercritical Fluid Technologies Inc., USA). Individual polymers or PMs were examined for an S-L transition temperature at 100 bar based on the observations reported by Pasquali et al.^24^ Approximately 2 mg of each polymer or PM was loaded into a melting-point capillary, which was secured in the sample holder attached to the lid of the high-pressure vessel. The vessel was closed and purged by opening both the entry and exit valves simultaneously for 5 min, allowing a continuous flow of CO_2_ to evacuate air. The exit valve was then closed, and the vessel was filled with liquid CO_2_ until the target pressure was achieved. The temperature was controlled by an integrated heating jacket, with adjustments (if required) made in increments not exceeding 2 °C during the experiment. The internal CO_2_ pressure was maintained at 100 bar by manually operating the piston throughout the test. The S-L transition was continuously monitored using a CCD camera mounted on a quartz viewing window of the vessel.

### 2.2 FDP-PEG Physical Mixtures (PMs) and Solid Dispersion (SDs)

FDP and PMs with the drug loadings of 10, 20, and 30% w/w were prepared from prescreened (450 μm mesh) materials. The necessary quantities (Table 1) of FDP and PEG 4K, 6K, 10K, and 20K were accurately weighed to obtain a total of 3 g of PMs. This was subsequently combined through the geometric mixing (a stepwise dilution method where small amounts of API are gradually blended with excipients to achieve uniform distribution) technique, followed by blending for 10 min at 34 rpm in a three-dimensional shaker mixer (TURBULA; T2 GE; WAB; Muttenz, Switzerland). The prepared samples were stored in glass vials at room temperature (23 ± 2 °C) and away from direct sunlight until further processing.

**Table 1:** Parameters for SD Preparation via scCO_2_ Processing

drug and polymer content					
s. no.	factor name	low	mid		high
1	FDP (% w/w)	10	20		30
2	PEG 4K, 6K, and 10K, 20K (% w/w)	90	80		70
scCO_2_ processing parameters					
3	temperature (°C) for PEG 4K, 6K, and 10K	45			
3*	*temperature (°C) for PEG 20K*	45	50	55	60
4	pressure (bar)	100			

SDs were prepared in static mode using a setup supplied by Thar Process Inc. (Pittsburgh, PA, USA). Three g of each PM was placed in a high-pressure vessel preheated to 45 °C, except for PEG 20K, which was also prepared at 50/55/60 °C. The vessel was then sealed, and liquid CO_2_ was introduced at a rate of 25 g/min until the target pressure of 100 bar was reached. The temperature and pressure were maintained for 1 h during which the mixtures were continuously agitated at 200 rpm to promote drug solubilization within the molten polymer. At the end of the experiment, the vessel was depressurized at a rate of 10 bar/min using a back pressure regulator. The prepared samples were stored in glass vials at ambient temperature and shielded from direct sunlight until required for subsequent analysis. The experimental process conditions are presented in Table 1.

### 2.3 Physicochemical Characterization of SDs

#### 2.3.1 Differential Scanning Calorimetry (DSC) Analysis

The DSC (DSC823e instrument, Mettler-Toledo, LLC in Leicester, UK) was used for the analysis of bulk FDP, scFDP, PEGs (4K, 6K, 10K, and 20K), PMs, and SDs. An accurately weighed (4 to 8 mg) sample was sealed in a 40 μL aluminum crucible and placed in the DSC sample loader. The analysis was performed under a constant nitrogen flow (50 mL/min) and between 25–160 °C at a heating rate of 10 °C/min. The DSC thermograms were obtained and integrated using Mettler-Toledo evaluation software.

#### 2.3.2 X-Ray Diffraction (XRD) Analysis

A Bruker D8 Advance diffractometer (Karlsruhe, Germany) was used to conduct XRD studies on bulk FDP, scFDP, PEGs (4K, 6K, 10K, and 20K), PMs, and SDs to evaluate the physical form of the raw materials. The study was conducted in the theta–theta reflection mode with a copper anode throughout the procedure. The samples were scanned over a 2-θ range of 2–60° using a slit width of 0.6 mm and a step size of 0.02°. The XRD integration software DiffracPlus and EVA V.14 were used for data gathering and analysis, respectively. XRD patterns were analyzed using a peak-area-based approach to estimate relative changes in crystallinity between samples. The integrated area of characteristic FDP diffraction peaks in the PMs and SDs was compared with that of the crystalline drug, which was defined as 100% relative crystallinity, allowing semiquantitative comparison of crystalline content across the processed samples.

#### 2.3.3 Attenuated Total Reflectance-Fourier-Transform Infrared (ATR-FTIR) Spectroscopy

The ATR-FTIR spectra of the drug, polymer, scCO_2_-processed drug, as well as the PMs and SDs, were obtained using a Spectrum Two ATR-FTIR spectrometer (PerkinElmer, UK). The crystal was first cleaned using ethanol, and background spectra were collected before the measurement. A small amount of each sample was placed directly onto the surface of a single-reflection horizontal ATR accessory equipped with a zinc selenide (ZnSe) crystal, and consistent contact between the sample and the crystal surface was ensured by applying uniform pressure using the built-in pressure arm. Spectra were acquired in ATR mode over the wavenumber range of 4000–400 cm^–1^ at a spectral resolution of 8 cm^–1^, with 16 scans averaged per spectrum. All measurements were performed under ambient conditions. The acquired spectra were processed using the Spectrum 10 software, with baseline correction, and were analyzed qualitatively to identify characteristic functional group vibrations and to assess potential drug–polymer interactions or structural changes induced by scCO_2_ processing.

#### 2.3.4 Scanning Electron Microscopy (SEM)

SEM SU8030 (Hitachi High-Technologies in Maidenhead, United Kingdom) micrographs were collected to determine the shape and surface morphology of the SDs, FDP, scFDP, and PEG, to ascertain the morphological alterations resulting from scCO_2_ processing. Approximately 2–5 mg of each sample was affixed to a stub using carbon adhesive, and the loose particles were removed before placing it in the instrument. The samples were then gold-coated (approximately 10 nm thickness), and the micrographs were collected at a voltage of 30.0 kV via the backscattered electron detection mode.

### 2.4 In Vitro Dissolution Studies

The dissolution of FDP equivalent to 10 mg from the PMs and SDs, as well as scFDP alone, was assessed utilizing the USP Type II paddle method (Hanson G2 Vision Classic 6, Chatsworth, Los Angeles, CA, USA). The SDs were sieved through a 450 μm mesh prior to the dissolution testing. The dissolution medium was chosen to be pH 6.5 phosphate buffer containing 1% sodium lauryl sulfate (SLS) as suggested in USP (Felodipine Extended-Release Tablets; USP-NF2025 Monograph). The samples were placed in 500 mL of dissolution buffer maintained at 37 ± 0.5 °C, and the study was performed at a paddle speed of 50 rpm. At designated time intervals of 5, 10, 15, 30, 45, and 60 min, 5 mL aliquots of the dissolution media were removed and substituted with an equivalent volume of fresh buffer. The samples were filtered using 0.45 μm PES syringe filters (Merck) and subsequently analyzed via ultraviolet–visible (UV–vis) spectroscopy (Cary 100 UV–visible spectrophotometer, Agilent Technologies, Cheadle, UK) at 364 nm against the dissolution media serving as a blank. The UV–vis analysis was also performed on placebo formulations to ensure no UV absorption was observed due to the polymer or tablet components. UV–vis analysis was preferred over the HPLC in this case because of its ease of operation. Moreover, a recent publication on rapidly dissolving FDP nanoparticle strips was also used as the basis for this decision, where they employed UV–vis analysis to quantify FDP at 364 nm.^16^

### 2.5 Orally Disintegrating Tablet (ODT) Preparation and Characterization

#### 2.5.1 ODT Preparation

The ODTs formulated in this study were composed of 15% w/w FDP (30% w/w drug-loaded SDs of PEG 4K and 20K). The tablets contained PEG as a carrier in SDs, microcrystalline cellulose (MCC) 102 as filler, croscarmellose sodium as a superdisintegrant, silicon dioxide as a flow aid, sucralose as a sweetener, orange flavor as a flavoring agent, and sodium stearyl fumarate (SSF) as a lubricant. The ODT composition is presented in Table 2.

**Table 2:** Composition of FDP (15% w/w) ODTs

ingredient	purpose	% w/w	mg/tab
**FDP-SD [PEG 4K or 20K]**	active	49	34
microcrystalline cellulose (MCC 102)	filler	32.5	23
croscarmellose sodium	super disintegrant	15	10.5
silicon dioxide	flow aid	0.5	0.4
sucralose	sweetener	1	0.7
orange flavor	flavouring agent	1	0.7
sodium stearyl fumarate	lubricant	1	0.7
**total**		**100.00**	**70**

All the excipients were sieved through a 450 μm mesh, followed by blending (except SSF), for 10 min at 34 rpm in a three-dimensional Shaker Mixer (TURBULA; T2 GE; WAB; Muttenz, Switzerland). After the blending, SSF was added to the blend and lubricated for 5 min. The final lubricated blend was assessed for precompression properties, such as bulk density, tapped density, Hausner ratio, and Carr’s index to ensure suitability for ODT manufacturing. The purpose of precompression studies is to ensure that the powder has the desired characteristics for subsequent processing.

#### 2.5.2 Flow Properties of SD Granules and ODT Blends

The bulk density of the sample was determined by gently filling the sample into a 10 mL graduated cylinder to approximately 50–60% of its volume. The weight of the powder was then determined, and the density was calculated by the mass-to-volume ratio. The tapped density was measured by subjecting the cylinder to 1250 taps over a period of 4 min, or until a constant volume was achieved. Using the obtained bulk and tapped density values, the flow properties of the sample were further evaluated by calculating Carr’s Index and Hausner’s Ratio. The purpose of precompression studies was to ensure that the powder mix had the desired flow characteristics for subsequent processing.

Flowability of powders was calculated using eqs 1–4

$$\mathrm{Bulk}\;\mathrm{density}\;g/\mathrm{mL}=\frac{\mathrm{Weight}}{\mathrm{Initial}\;\mathrm{volume}}$$ 1

$$\mathrm{Tapped}\;\mathrm{density}(g/\mathrm{mL})=\frac{\mathrm{Weight}}{\mathrm{Final}\;\mathrm{volume}}$$ 2

$$\mathrm{Compressibility}\;\mathrm{index}=\frac{\mathrm{Tapped}\;\mathrm{density}-\mathrm{Bulk}\;\mathrm{density}}{\mathrm{Tappe}\;\mathrm{density}}\times 100$$ 3

$${\mathrm{Hausner}}^{\prime }s\;\mathrm{ratio}=\frac{\mathrm{Tapped}\;\mathrm{density}}{\mathrm{Bulk}\;\mathrm{density}}$$ 4

#### 2.5.3 Tablet Compression and Evaluation

ODTs were compressed using Roltgen marking systems, Flexitab Trilayer automated tablet compression (Germany) instrument with 6 mm round flat-faced lower punch and bevel-edge upper punch faces. Tablets were compressed at a fill volume/depth of 3.5 mm and a compression force of 10 ± 3 kN. The ODTs manufactured in this study contained 15% w/w FDP per tablet and were formulated using only the 30% w/w drug-loaded SDs of PEG 4K and 20K.

The physicochemical characterization of the tablets was conducted in accordance with USP guidelines to evaluate their suitability as a drug delivery system. Weight variation was assessed using a laboratory weighing balance (Mettler Toledo; XS105 dual-range balance) with a sample size of 20 tablets. Hardness testing was performed on ten tablets using a tablet hardness tester (Dr Schleuniger Pharmatron 5Y), while thickness was measured for ten tablets using a digital vernier calliper (Aickar). Disintegration time was determined for six tablets employing a USP disintegration apparatus (Electrolab; ED-2L). Additionally, content uniformity was evaluated for ten tablets, and in vitro dissolution studies were carried out on three tablets. An identical dissolution procedure was then performed on the tablets, substituting the SD samples with ODTs to evaluate the FDP release from the prepared oral solid dosage forms.

### 2.6 Stability Study

A short-term (30 days) stability study was conducted in accelerated (40 °C/75% RH) and ambient conditions on PEG 4K and 20K SDs and corresponding ODTs. At 30-day time points, the drug dissolution in 60 min was measured using the method detailed in Section 2.4 and compared with the drug release at day 0.

## 3 Results and Discussion

### 3.1 S-L Transition and SD Preparation

A study by Pasquali et al. reported melting point (*T*_m_) depression of PEG 4K in scCO_2_, showing an initial linear decrease in Tm with increasing pressure, reaching approximately 45 °C at 100 bar, beyond which no further reduction was observed.^24^ Based on this observation, Tm determination in the present study was also performed at 100 bar, with the gradual increase in temperature until a clear S-L transition was observed. At 100 bar, PEG 4K, 6K, and 10K exhibited a complete phase transition (S-L) at ∼45 °C, whereas PEG 20K showed the onset of melting at around 50 °C and achieved complete S-L transition at ∼60 °C. The FDP did not exhibit any observable S-L transition under the investigated pressure and temperature in scCO_2_. A visual representation of these transitions is provided in Figure 2.

**Figure 2:**
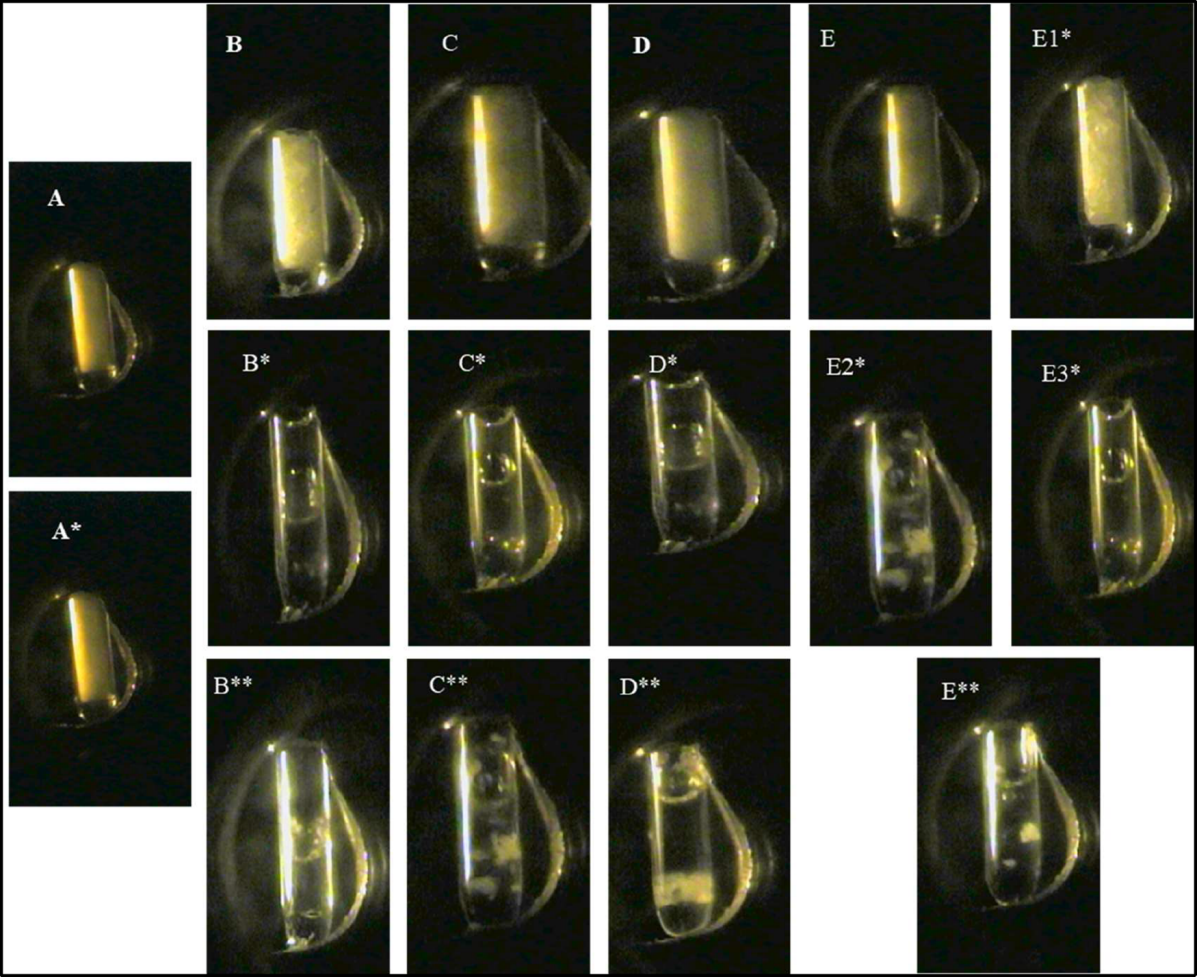
S-L transition of drug, PEG, and physical mixtures at 100 bar in scCO_2_. [(A–E) Samples at atmospheric pressure: A (FDP), B (PEG 4K), C (PEG 6K), D (PEG 10K), and E (PEG 20K). (A*–E*) Samples in scCO_2_ at 45 °C, except PEG 20K, where E1*, E2*, and E3* represent observations at 45, 50, and 60 °C. FDP (A*) remained in the solid-state under these conditions. (B**–E**) Physical mixtures (30% w/w FDP) in scCO_2_ at 100 bar and 45 °C, except PEG 20K, which was examined at 60 °C.].

The *T*_m_ depression of a polymer in scCO_2_ is generally attributed to a combination of plasticization, reduced lattice stability, and phase-equilibrium effects.^27^ The scCO_2_ acts as a plasticizer when it is absorbed in the amorphous regions of a semicrystalline polymer, leading to thermodynamic instability of the crystalline domains and resulting in a decrease in *T*_m_.^24^ The CO_2_ molecules also lower the cohesive energy density by intercalating at the interfaces of crystalline lamellae, which reduces the energy required to disrupt the crystalline lattice.^28^ In addition, scCO_2_ behaves as a diluent in the melt phase, so the equilibrium between crystalline and molten phases is reached at a lower temperature.^29^ The extent of *T*_m_ depression depends on polymer crystallinity and diffusivity. For example, semicrystalline polymers like PEGs have loosely packed crystalline regions, and they exhibit high CO_2_ sorption capacity, which enhances polymer-scCO_2_ interactions and results in Tm depression.^30^

PMs with 30% w/w FDP displayed S-L transitions comparable to those of the corresponding PEGs, indicating that the melting was primarily governed by the polymer. PEG contains hydroxyl (−OH) groups capable of donating hydrogen bonds, as well as ether oxygen atoms that can act as hydrogen-bond acceptors. FDP, in turn, contains hydrogen-bond donor sites (−NH group within the dihydropyridine ring) and hydrogen-bond acceptor sites (carbonyl groups within the ester functionalities). This can promote complementary interactions and mixing of FDP within the molten PEG phase. The scCO_2_-induced plasticization of PEGs results in reduced melting temperature and viscosity. FDP has only limited solubility in scCO_2_, but the combined effects of polymer melting and plasticization facilitate the dispersion of FDP within the PEG matrix via the hydrogen-bond-mediated drug–polymer interactions. The homogeneous dispersion of FDP in the polymer matrix can also be expected to enhance dissolution rate and apparent solubility.^7,31^

Solid dispersions (SDs) were prepared by the organic-solvent-free scCO_2_ processing of FDP-PEG PMs. In general, the formation of SDs involves three key stages: (i) generation of a polymer-rich molten or plasticized phase, (ii) intimate mixing and dispersion of the drug within this molten phase, and (iii) solidification upon depressurization and cooling.^31^ This study also aimed to understand if the effective SD formation required both the drug and the polymer to be above their S-L transition temperature, or whether polymer melting alone was sufficient. Therefore, experiments were conducted at pressures and temperatures at/above the S-L transition of the polymer, but not that of the drug. FDP-PEG 4K, 6K, and 10K SDs were prepared at 100 bar and 45 °C based on the S-L transition temperature results. The FDP-PEG 20K SDs prepared at 45 °C and 100 bar were not expected to result in SD formation due to PEG 20K’s high S-L temperature. Hence, further studies at 50, 55, and 60 °C were also conducted to understand the impact of the temperature increase on SD formation.

### 3.2 DSC Analysis

DSC analysis was performed to investigate the physical state of FDP within the polymer matrix after scCO_2_ processing. Thermograms for FDP, PEG, PMs, and SDs prepared with PEG 4K are presented in Figure 3. The FDP and scCO_2_-treated FDP (not presented) both displayed a melting peak at approximately 148–150 °C, due to the drug’s unique crystalline structure. This confirmed that scCO_2_-processing of the drug on its own does not cause any changes to its morphological and crystalline characteristics. The PEG 4K, 6K (Figure S1) and 10K (Figure S2), exhibited a distinct melting peak at ∼62 °C, while PEG 20K displayed a sharp melting peak at 65.5 °C (Figure S3). PEG, being a semicrystalline polymer, demonstrated slight differences in melting peaks based on its molecular weight.

**Figure 3:**
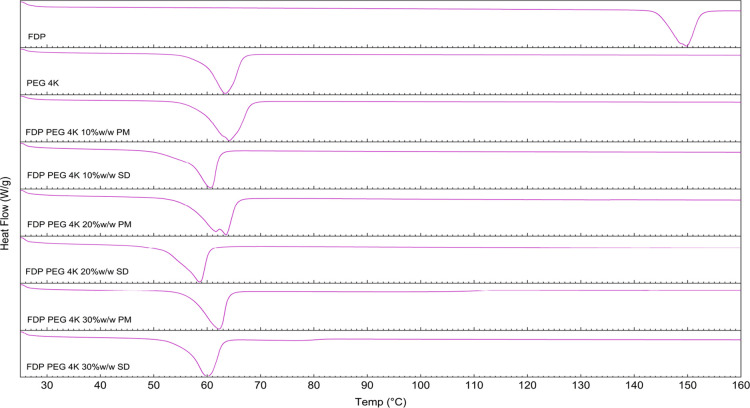
DSC thermograms of bulk FDP, PMs, and SDs with PEG 4K.

Across all PMs (10, 20, and 30% w/w FDP), the PEG melting transition remained relatively unchanged in both onset and peak temperature; there was no appreciable shift or reduction observed for PEG in PMs. This indicates that the simple blending of PEG and FDP does not promote a molecular-level interaction. In contrast, the SDs demonstrated melting point depression of PEGs alongside peak broadening, indicating mixing at the molecular-level and disruption of PEG’s semicrystalline lattice due to incorporation of FDP molecules within amorphous or partially amorphous PEG domains. This observation is consistent with the impurity-induced crystallinity suppression associated with the crystalline order disruption, leading to the reduction in lattice stability.^32^ The depression in the melting point was higher in SDs prepared with lower-molecular-weight PEGs. FDP-PEG 20K formulations showed minimal change in melting temperature for both PM and SD, at 45 °C (Figure S3). Hence, the processing temperature was increased to determine if that improved SD formation (Figure 4).

**Figure 4:**
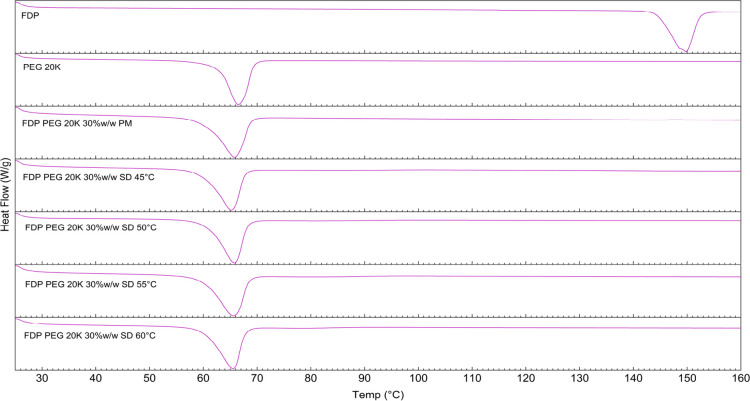
DSC thermograms of bulk FDP, PMs, and SDs with PEG 20K at different processing temperatures.

The impact on the melting temperature of PEG 20K at the higher processing temperature (50–60 °C) was also limited, which could be due to the higher crystallinity or lamellar integrity of the polymer that could not be significantly disrupted by the FDP molecule. The drug may be preferentially located in amorphous regions rather than being incorporated into the polymer crystals themselves. Nonetheless, DSC analysis of both PMs and SDs suggests that PEG is capable of solubilizing FDP, and scCO_2_-processing aids the intermolecular interaction, which may not be achievable by simple mixing of two components.

### 3.3 XRD Analysis

XRD analysis was conducted to assess changes in the crystalline nature of bulk FDP, scFDP and to compare drug crystallinity in PMs and SDs. The diffractogram of bulk FDP confirmed its crystalline nature, with characteristic diffraction peaks observed at 2θ values of 10.5, 11, 20.5, 23.5, 24.5, 25.5, and 26.5°. The FDP peaks were also present in scFDP, confirming that the scCO_2_ processing of the drug alone does not change the drug’s crystallinity. PEG diffractograms had two prominent reflections at approximately 19 and 23.5°, confirming its semicrystalline structure. Figure 5 presents the X-ray diffractogram of bulk FDP, scFDP, PMs, and SDs prepared with PEG 4K. Diffractograms of SDs prepared with PEG 6K, 10K and 20K processed at 45 °C are presented in Figures S4–S6, respectively.

**Figure 5:**
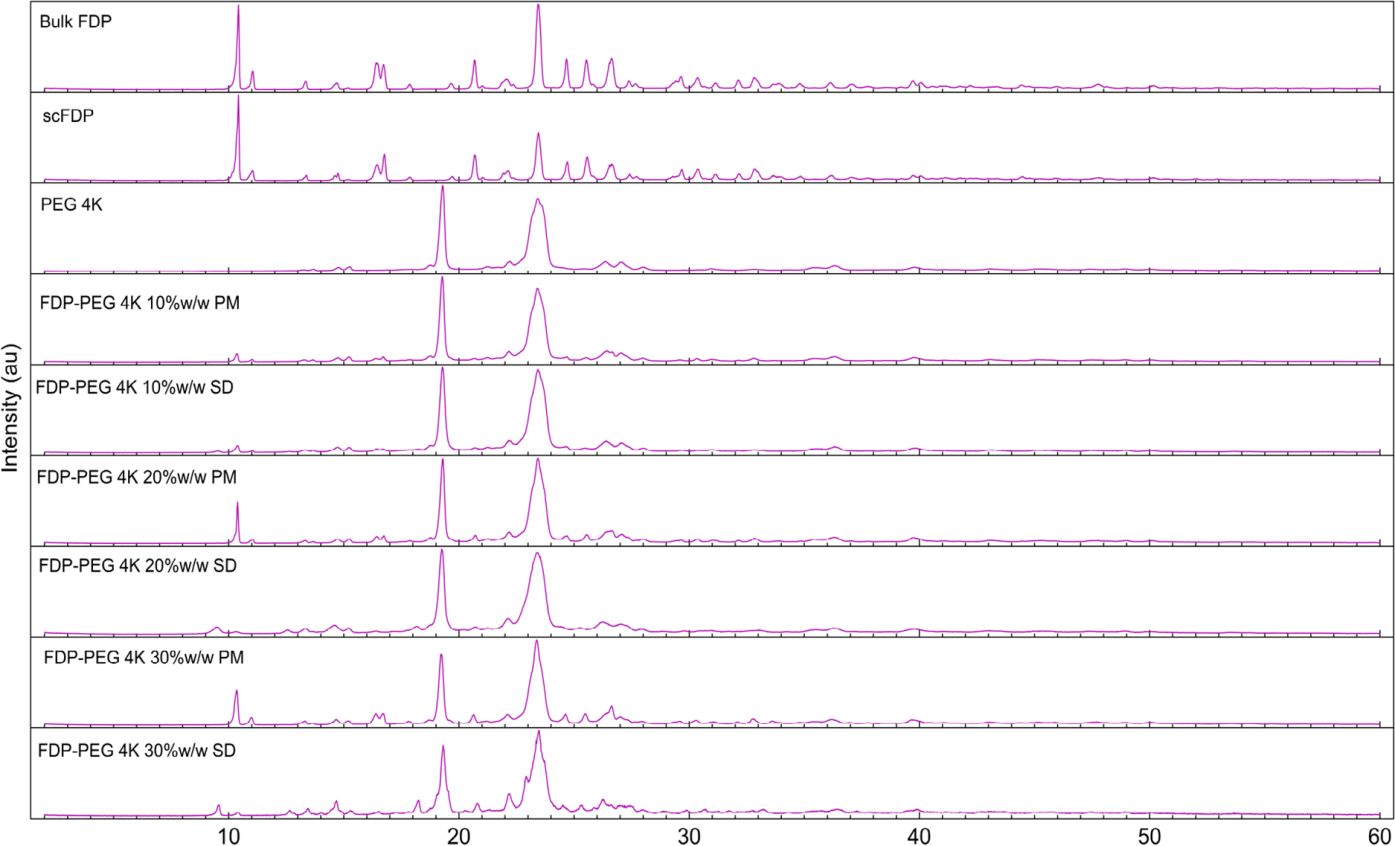
XRD diffractogram of bulk FDP, scFDP, and SDs prepared with PEG 4K.

The PMs retained the semicrystalline characteristics of both components, largely displaying superimposed diffraction peaks of FDP and PEG, confirming that simple physical blending did not induce significant disruption of the drug crystal lattice. In contrast, the SDs exhibited reductions in the intensity and sharpness of FDP diffraction peaks, particularly at higher polymer contents and lower PEG molecular weights. The FDP peaks were notably reduced, especially in SDs prepared with lower drug loadings and low-molecular-weight PEG. There was some peak broadening in SDs relative to the PMs, indicative of partial amorphization of FDP within the polymer matrix. This effect was more pronounced for SDs prepared with lower-molecular-weight PEGs, probably attributed to enhanced polymer chain mobility and more efficient drug–polymer mixing.

For PEG 20K systems (Figure 6), this also resulted in peak suppression and broadening, with the greatest reduction observed at 60 °C. The dependence of temperature on subtle differences of FDP peaks in diffractograms could be due to PEG 20K’s higher S-L transition temperature and melt viscosity, which will require higher temperatures to facilitate effective drug dispersion within the matrix.

**Figure 6:**
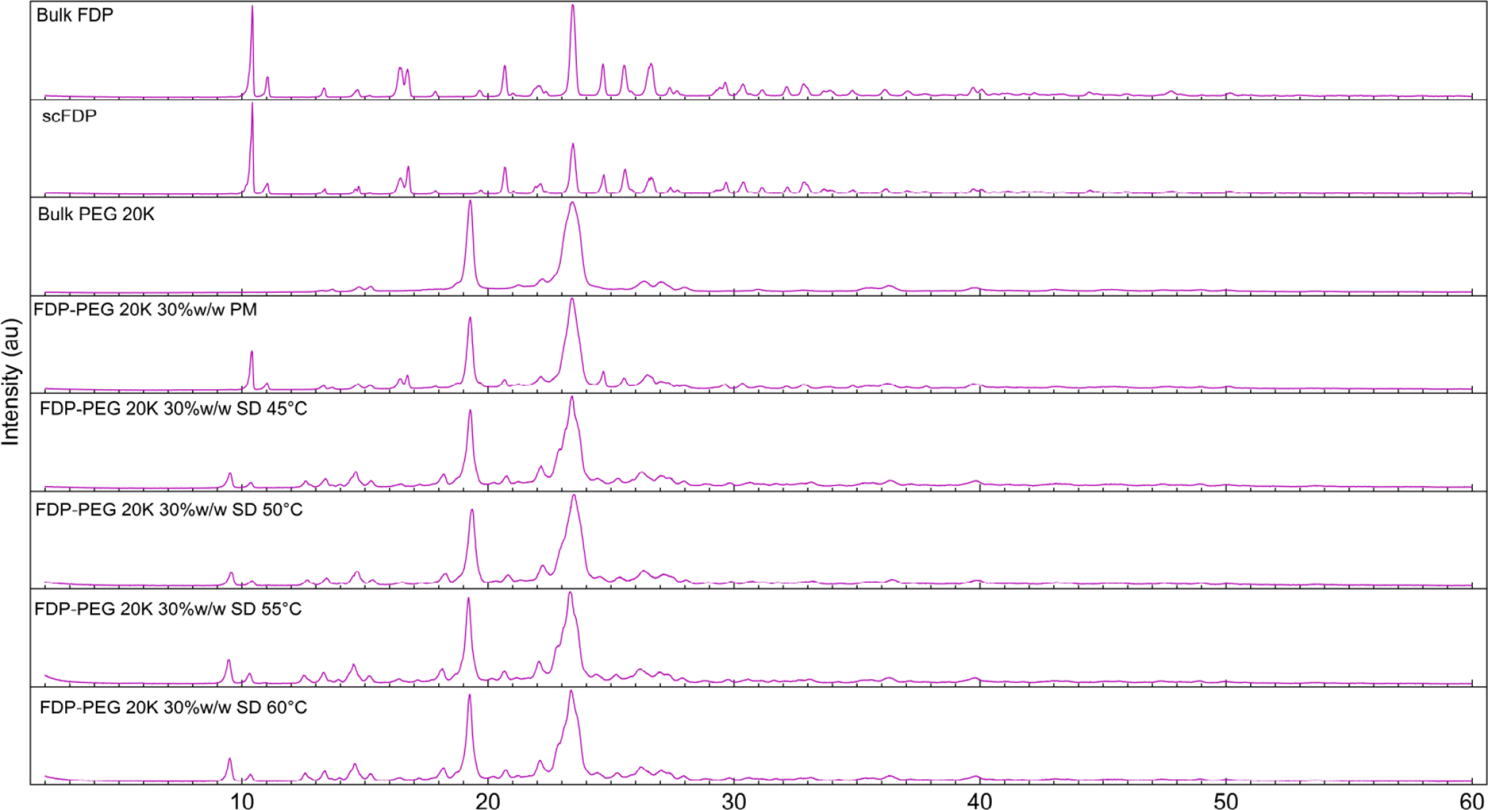
XRD diffractograms of bulk FDP, PMs, and SDs with PEG 20K at different processing temperatures.

Overall, the XRD data indicate a reduction in FDP crystallinity in the SDs prepared under optimized scCO_2_ processing conditions. Still, it is also evident from the diffractograms that the complete amorphization of FDP did not occur, even though DSC suggested that complete miscibility at the molecular-level is possible, as shown by the disappearance of the FDP melt peak. This may also indicate that further optimization of the processing parameters may be required with respect to temperature and pressure. Nevertheless, the presence of some drug crystallinity in an SD may not necessarily translate into suboptimal dissolution performance.^33^ Hence, further analysis was performed on these systems to understand if expected dissolution improvement could be achieved using PEGs as polymeric carriers and scCO_2_ as the processing method to prepare the SDs.

### 3.4 ATR-FTIR Spectroscopy

ATR-FTIR spectroscopy was employed to understand potential molecular interactions and solid-state changes in FDP, scFDP, PEGs, PMs, and SDs prepared via scCO_2_ processing. Figure 7 presents the ATR-FTIR spectrum of PEG 4K SDs along with the drug. The spectra for PEG 6K, 10K, and 20K are presented in Figures S7–S9, respectively.

**Figure 7:**
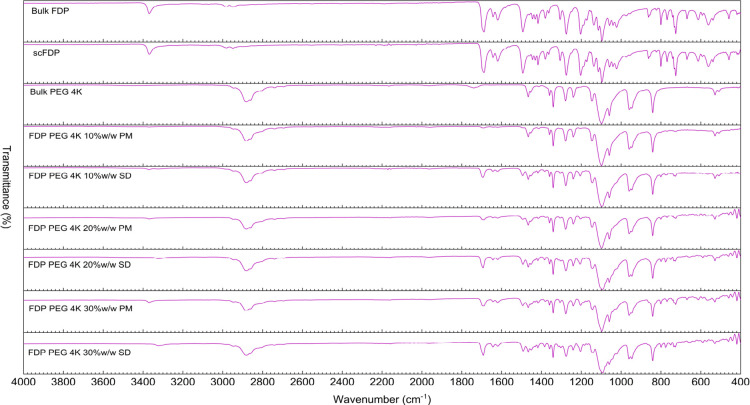
ATR-FTIR spectra of bulk FDP, scFDP, PMs, and SDs prepared with PEG 4K.

The bulk FDP exhibited characteristic absorption bands corresponding to its functional groups, including the N–H stretching vibration of the dihydropyridine ring in the region of ∼3300–3400 cm^–1^, strong ester carbonyl (C=O) stretching bands around ∼1700–1725 cm^–1^, and multiple fingerprint-region bands associated with aromatic and ester functionalities. The spectrum of scFDP closely resembled that of bulk FDP, indicating that exposure to scCO_2_ alone did not induce chemical degradation or significant structural modification of the drug.

PEG 4K and PEG 20K displayed characteristic absorption bands, including a broad O–H stretching band centered around ∼3400 cm^–1^, C–H stretching vibrations near ∼2880 cm^–1^, and prominent C–O–C stretching bands in the region of ∼1100–1140 cm^–1^, consistent with their polyether structure.

The PMs and SDs prepared with PEG 4K exhibited subtle spectral changes but remained relatively unchanged. There were subtle broadening and slight attenuation of the FDP N–H stretching band and minor changes in the intensity and definition of the ester carbonyl stretching region. These changes suggest weak intermolecular interactions between the drug and polymer, possibly via hydrogen bonding. Importantly, no new absorption bands were observed, confirming that no covalent interactions or chemical transformations occurred during scCO_2_ processing.

ATR-FTIR spectroscopy results for SDs prepared with PEG 20K at various temperatures are presented in Figure 8. In general, similar trends were observed, although the spectral changes with respect to peak broadening and attenuation of FDP-related peaks, suggestive of noncovalent type interactions between the drug and PEG 20K.

**Figure 8:**
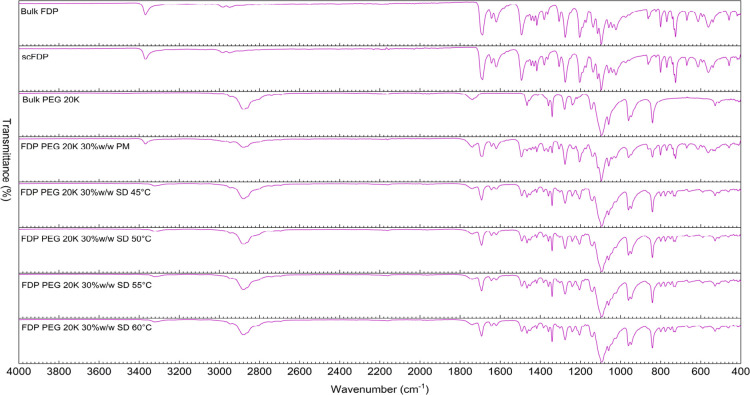
ATR-FTIR spectra of bulk FDP, scFDP, PMs, and SDs prepared with PEG 20K at different processing temperatures.

Overall, the ATR-FTIR data indicate that scCO_2_-assisted processing promoted mixing of FDP with PEG, leading to weak, noncovalent drug–polymer interactions. While ATR-FTIR alone cannot confirm complete amorphization, the band broadening and attenuation, together with the absence of new peaks, are consistent with partial or substantial disruption of the crystalline drug structure.^34,35^ These findings, along with the observations from the XRD and DSC data, demonstrate the desired mixing between the drug and polymer that results in the reduction of FDP crystallinity in the SDs prepared via scCO_2_ processing.

### 3.5 SEM Analysis

The surface morphology of bulk FDP, scFDP, PEGs, and SDs was examined using SEM, and representative micrographs are shown in Figure 9. Bulk FDP (Figure 9A) consisted predominantly of well-defined plate-like and elongated crystalline particles with sharp edges and smooth faces, characteristic of a highly crystalline material. The morphology of scFDP (Figure 9B) was comparable to that of bulk FDP, indicating that the scCO_2_ processing under the investigated conditions did not induce significant changes in crystal habit or surface characteristics.

**Figure 9:**
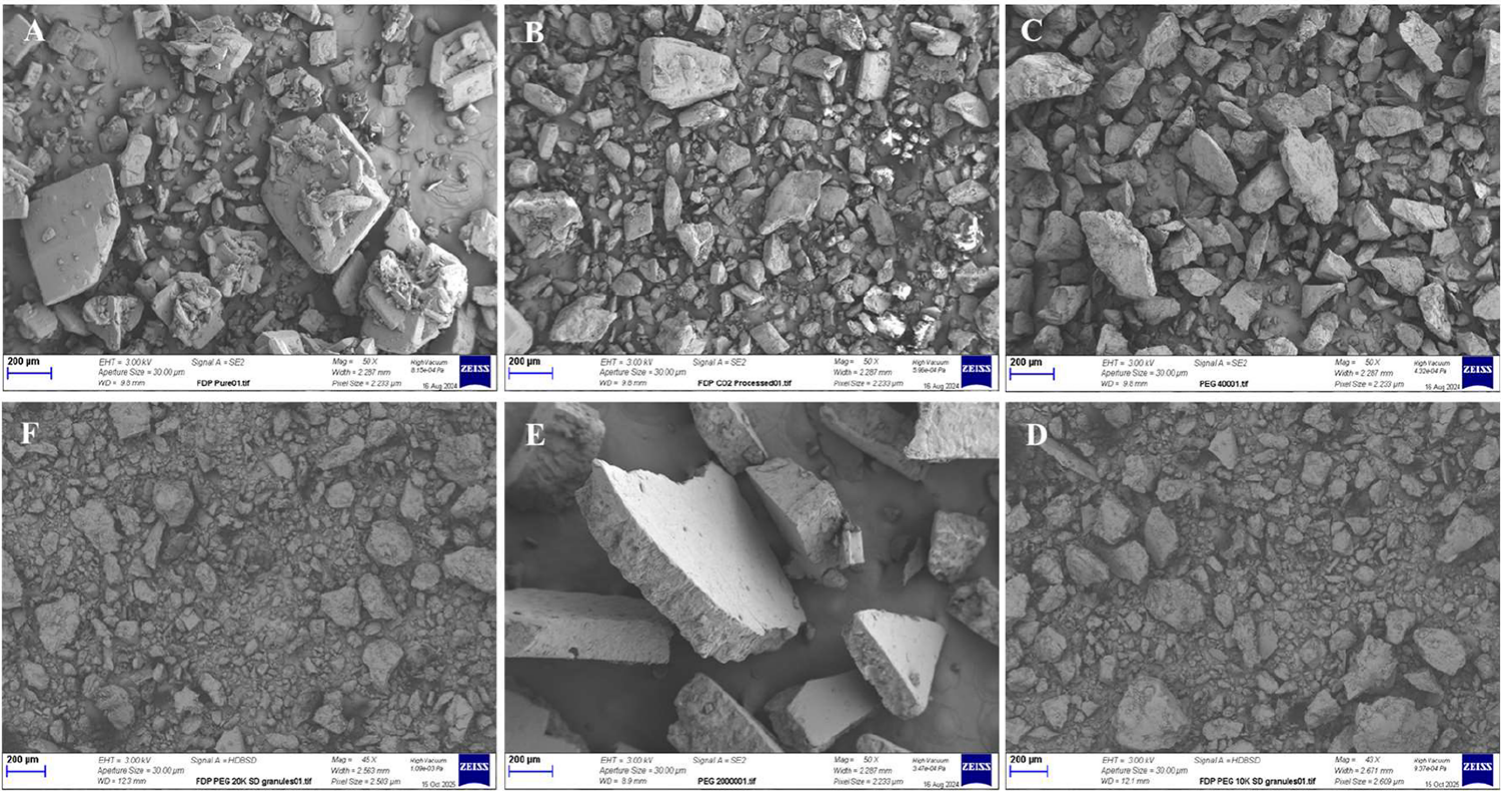
SEM images of (A) bulk FDP, (B) scFDP, (C) PEG 4K, (D) PEG 4K 30% w/w SD, (E) PEG 20K, and (F) PEG 20K 30% w/w SD.

PEGs exhibited irregular, angular particles with fractured surfaces and an absence of well-defined crystalline facets, consistent with their semicrystalline polymeric nature, as shown in Figure 9C (PEG 4K) and E (PEG 20K).

In contrast, the SDs prepared via scCO_2_-processing (Figure 9D,F) displayed a markedly different morphology, consisting of irregular, polymer-like granules with rough surfaces and no clearly discernible FDP crystals. The loss of the characteristic plate- and needle-like morphology of FDP suggests that the drug was embedded within or coated by the polymer matrix following scCO_2_-assisted processing. All SD formulations, irrespective of PEG molecular weight or drug loading, exhibited comparable surface morphology, and images presented are therefore representative.

### 3.6 In Vitro Dissolution Studies of Bulk FDP, scFDP, PMs, and SDs

Figure 10 illustrates the dissolution behavior of FDP from the formulated SDs prepared with PEG 4K, 6K, 10K and 20K with varying drug loadings (10, 20, and 30% w/w), and compares it against bulk FDP, scFDP, and the corresponding PMs.

**Figure 10:**
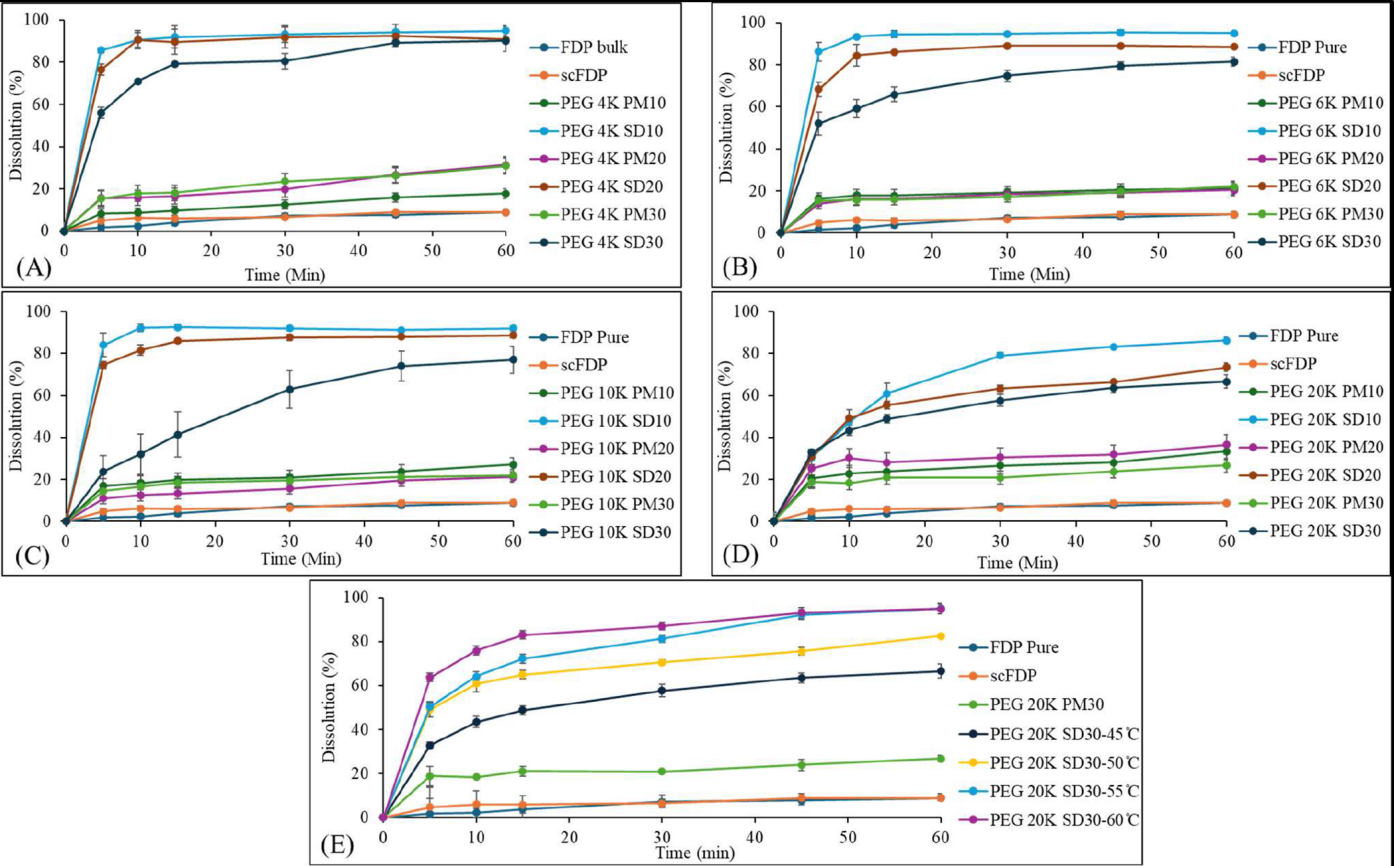
In vitro dissolution profiles of bulk FDP, scFDP, PMs, and SDs; (A) PEG 4K, (B) PEG 6K, (C) PEG 10K, (D) PEG 20K, and (E) PEG 20K at various temperatures.

Bulk FDP and scFDP exhibited minimal dissolution throughout the test period, consistent with the poor aqueous solubility and high crystallinity of FDP. The PMs showed only marginal improvement relative to bulk FDP, confirming that simple physical blending with hydrophilic polymers is insufficient to overcome the dissolution limitations of crystalline drugs. In contrast, all SDs exhibited markedly enhanced dissolution rates, highlighting that SD formation was necessary for the dissolution enhancement of FDP. This behavior is consistent with other scCO_2_-based studies demonstrating that solvent-free polymer-mediated SDs significantly improve drug dissolution.^31,36^ Across all tested formulations, the dissolution profiles clearly demonstrated that polymer content and drug loading had a strong influence on dissolution performance.

In general, formulations with higher PEG content achieved faster and higher drug dissolution, with the 10% w/w FDP containing SDs consistently outperforming, irrespective of the molecular weight of PEG. The dissolution improvement was due to a combination of reduced FDP crystallinity, improved wettability, and more homogeneous drug dispersion within the polymer matrix. The mean percentage dissolution of FDP from various PMs and SDs is summarized in Table 3.

**Table 3:** Mean Percentage Dissolution at 10, 30, and 60 min from Various PMs and SDs

SD with 10% w/w FDP											
	PEG 4K		PEG 6K		PEG 10K		PEG 20K				
dissolution at 10, 30, and 60 min	PM (%)	SD (%)	PM (%)	SD (%)	PM (%)	SD (%)	PM (%)	SD-45 °C (%)	SD-50 °C (%)	SD-55 °C (%)	SD-60 °C (%)
*D*_10_	9	91	18	93	18	92	22	46	not determined		
*D*_30_	13	93	19	95	21	92	26	79			
*D*_60_	18	95	21	95	27	92	33	86			
**SD** with 20% w/w FDP											
*D*_10_	16	91	16	45	13	82	30	49	not determined		
*D*_30_	20	92	18	89	16	88	31	63			
*D*_60_	31	91	20	89	21	90	37	73			
**SD** with 30% w/w FDP											
*D*_10_	16	71	16	59	17	32	18	44	61	64	76
*D*_30_	19	81	17	75	20	63	21	57	71	81	87
*D*_60_	25	90	22	81	22	77	27	67	83	95	95

The rapid FDP dissolution (5–10 min) is predominantly polymer-controlled, which is characteristic of amorphous or molecularly dispersed drug systems stabilized by hydrophilic polymers.^37,38^ The reduction in dissolution rate with increasing drug loading can be rationalized by the finite solubilization and stabilization capacity of the polymer matrix. At lower drug loadings (10% w/w), the high polymer-to-drug ratio ensures effective wetting, molecular dispersion, and stabilization of FDP within the PEG matrix. As drug loading increases to 20–30% w/w, the relative polymer content decreases, limiting the ability of PEG to fully solubilize and stabilize the drug. Although PEG does not form classical micelles, it can generate transient micelle-like or coil-based solubilizing domains in aqueous media that facilitate the dissolution of hydrophobic drugs.^15,34^ At lower drug loadings, the higher PEG content provides a greater number of such solubilizing domains, enabling efficient partitioning of FDP into the aqueous phase, leading to rapid dissolution.^39^ The increase in drug content and simultaneous decrease in polymer in the SD limit the number of available solubilizing domains and reduce the capacity of PEG to accommodate the dissolved drug.^40^ Consequently, polymer-assisted solubilization becomes saturated, leading to slower dissolution and reduced maintenance of supersaturation at higher drug loadings.

The processing temperature played an important role for PEG 20K SDs, where FDP showed the fastest dissolution from the samples processed at 60 °C compared with SDs prepared at 45, 50, or 55 °C. Similar temperature-driven improvements have been reported for scCO_2_-processed SDs, where higher processing temperatures resulted in lower drug crystallinity and higher dissolution.^26,31^ This improvement could be attributed to enhanced polymer plasticization, increased molecular mobility resulting in improved drug–polymer mixing at temperatures above the S-L transition of the polymer.

Overall, these findings confirm that SDs of FDP in various PEGs can be prepared at comparatively low temperatures, which leads to enhanced drug dissolution. However, the resultant rate of drug dissolution can be dependent on various factors, including the polymer, drug loading and processing parameters.

### 3.7 Powder Blend and Tablet Evaluation

#### 3.7.1 Flow Properties of the Powder Blends

Table 4 contains the flow properties data on FDP and the ODT powder blends. The FDP itself had poor flow properties as evidenced by a high Carr’s index (>20) and Hausner ratio (>1.25), possibly due to its crystalline nature. The PEG 4K and 20K SDs showed fair-to-good flow with a CI of ∼15 and an HR of ∼1.2. The ODT blend of FDP-PEG 4K showed an improvement in the flow properties (CI ∼ 13.8, HR ∼ 1.2) in comparison to the drug alone and other SDs.

**Table 4:** Flow Properties of SDs and ODT Blends

	flow properties			
drug/powder blend	bulk density (g/mL)	tapped density (g/mL)	Carr’s index (CI)	Hausner’s ratio (HR)
FDP	0.41	0.53	22.6	1.3
PEG 4K 30% SD (45 °C)	0.68	0.81	16.0	1.2
PEG 20K 30% SD (60 °C)	0.78	0.92	15.2	1.2
4K ODT blend before blending	0.61	0.75	18.7	1.2
4K ODT blend after blending	0.69	0.8	13.8	1.2
20K ODT blend before blending	0.68	0.95	28.4	1.4
20K ODT blend after blending	0.76	0.96	20.8	1.3

The PEG 20K had comparatively poor flow properties with the CI of 20.8 and HR of 1.3. Both ODT blends with PEG 4K and 20K SDs showed an improvement in flowability after blending. Although the blend prepared with PEG 20K resulted in slightly higher than the desired CI and HR values, it could still be considered acceptable for tablet compression.^41^

#### 3.7.2 Compressed Tablets Properties

The physical evaluation of FDP-PEG 4K and 20K ODT formulations demonstrated compliance with USP pharmacopeial specifications (905 Uniformity of Dosage Units, 701 Disintegration, 1216 Tablet Friability, 1217 Tablet Breaking Force, and 711 Dissolution) for weight variation, thickness, hardness, disintegration, and friability. Both formulations exhibited average tablet weights within the acceptable ±10% range of the target 70 mg. Tablets were uniform in size and shape, with a consistent diameter of 6 mm and a thickness between 2.6 and 2.8 mm. The hardness of the 20K ODT (25–32 N) was higher than that of the 4K ODT (20–22 N), reflecting greater mechanical strength. ODT characteristics are summarized in Table 5.

**Table 5:** Compressed Tablet Properties

		actual weight		
measured parameters	target weight (±10%) mg	min wt. (mg)	max wt. (mg)	avg. wt. (mg)
4K ODT	70 mg (63–77)	65	76	71
20K ODT		64	74	69

parameter	4K ODT		20K ODT	
diameter/appearance	6 mm/round with a flat bottom, and white to off-white tablet			
thickness (mm)	2.6–2.8			
hardness (N)	20–22	25–32		
disintegration (s)	18	22		
friability (%)	1.2	0.45		

Despite these differences, both formulations disintegrated rapidly within the pharmacopoeial limit of 30 s, with the 4K ODT showing a slightly faster disintegration (18 s) compared to the 20K ODT (22 s). Friability testing revealed that the 4K ODT exceeded the 1% threshold (1.2%), suggesting lower abrasion resistance, whereas the 20K ODT showed excellent robustness with a friability of 0.45%. Overall, the 20K ODT exhibited superior mechanical properties and acceptable disintegration behavior, making it better suited for further development.

#### 3.7.3 In Vitro Dissolution Studies for ODTs

ODTs were successfully formulated using the highest drug-loaded SDs (30% w/w FDP) prepared with PEG 4K and PEG 20K. The ODT formulations exhibited rapid and extensive drug release, closely matching the dissolution behavior of FDP from their corresponding SDs. In both PEG 4K and PEG 20K systems, the ODTs achieved 70–80% drug release within the first 10 min, followed by a gradual approach to 100% within 1 h, as presented in Figure 11.

**Figure 11:**
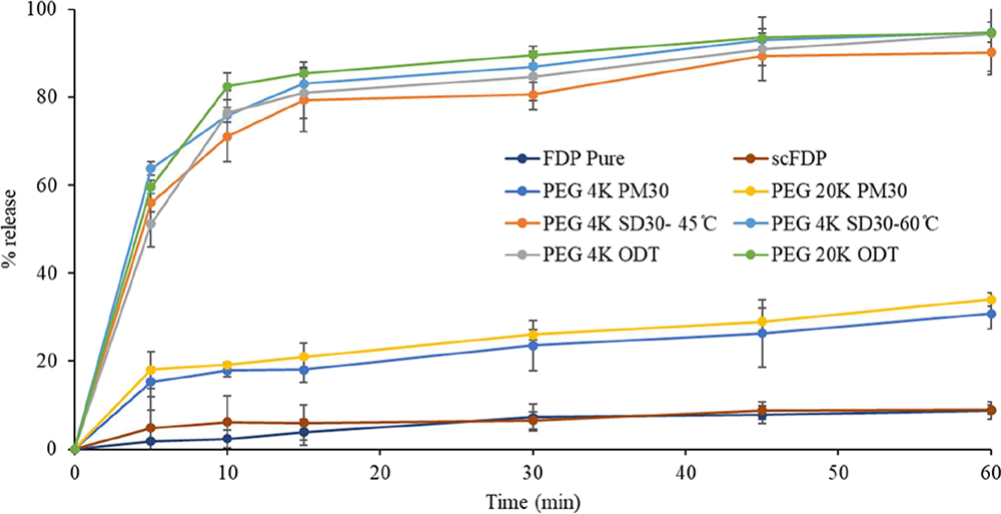
FDP dissolution from ODTs prepared using PEG 4K and PEG 20K SDs with 30% w/w drug loading.

There was no reduction in the rate or extent of FDP dissolution following compression of SDs into ODTs, indicating that the tabletting process did not compromise the SD characteristics responsible for enhanced dissolution. These results demonstrate that ODT manufacture using SDs prepared by scCO_2_-processing can be an effective method to develop dissolution-enhanced patient-friendly dosage forms as a practical means of improving the oral delivery of poorly water-soluble drugs while simultaneously addressing administration challenges in patient populations with swallowing difficulties.

### 3.8 Stability Study

A short-term stability study was carried out under ambient and accelerated conditions. The dissolution study showed 94.8 ± 3.5, 90.9 ± 2.3, 94.7 ± 4.8 and 94.4 ± 3.2% drug release from PEG 20K, 4K SDs, and PEG 20K, 4K ODTs, respectively. The drug release after 30-day (Table S1) storage at both the ambient and accelerated conditions remained unchanged. A significant challenge of SDs is the recrystallization of amorphous drugs, but polymeric carriers can prevent that by inhibiting the molecular mobility of the drug. If recrystallization happens, then the improvement of drug dissolution would be compromised. The short-term stability study showed no significant change in drug dissolution while stored in different conditions. However, a full-scale stability study in appropriate conditions per the ICH guidelines should be carried out to verify this.

## 4 Conclusions

In this study, a solvent-free scCO_2_-based approach was successfully employed to prepare SDs of FDP, resulting in significantly enhanced drug dissolution across all investigated drug–polymer ratios. The scCO_2_ processing parameters, FDP-to-PEG ratio, and polymer molecular weight influenced the formulation properties. Solid-state characterization by DSC and XRD confirmed a reduction in FDP crystallinity, indicative of at least partial amorphization. The developed SDs were subsequently incorporated into ODTs using FDP-PEG 4K and FDP-PEG 20K SDs with 30% w/w drug loading. The ODTs retained the enhanced dissolution characteristics of the parent SDs, demonstrating that tablet compression did not adversely affect the solid-state properties or dissolution behavior of FDP. However, differences in mechanical performance were observed between the formulations. ODTs prepared using FDP-PEG 4K SDs exhibited friability values exceeding 1%, accompanied by noticeable edge erosion during friability testing, whereas ODTs prepared with FDP-PEG 20K SDs showed acceptable mechanical robustness, with low friability and minimal surface damage.

Based on the combined dissolution and mechanical performance, ODT containing FDP-PEG 20K was found to be the most suitable, offering enhanced drug dissolution alongside adequate tablet integrity. Overall, this work demonstrates the potential of scCO_2_-assisted processing as a green, solvent-free, and potentially scalable strategy for the development of SD-based oral dosage forms. The successful integration of SDs into ODTs highlights a promising pathway for improving the delivery of poorly water-soluble drugs while supporting environmentally sustainable practices.

## Supplementary Material



## Notes

Stability studies of SDs and ODTs (Table S1); and DSC thermograms, XRD diffractograms, and ATR-FTIR spectra of bulk FDP, scFDP, PMs, and SDs prepared with PEG 6K, 10K, and 20K (Figures S1–S9) (DOCX)
