# Supplementary material for: Preparation of Felodipine–PEG Solid Dispersions by Solvent-Free scCO2 Processing and Their Translation into Orally Disintegrating Tablets
Source: ACS Omega. 2026 Feb 12;11(7):12766–78. doi: 10.1021/acsomega.5c13416 (PMC12947192; doi:10.1021/acsomega.5c13416)
Supplement: Supplementary file 1 [file ao5c13416_si_001.docx]

**Supplementary material**

Preparation of Felodipine-PEG Solid Dispersions by Solvent-Free scCO_2_ Processing and their Translation into Orally Disintegrating Tablets

Siva S. Kolipaka^1^, Laura A. Junqueira^2^, Elizabeth J. Pathrapankal^4^, Dennis Douroumis^3^, Vivek Trivedi^4*^

^1^ Centre for Research Innovation, University of Greenwich, Medway Campus, Chatham Maritime, ME4 4TB, UK

^2^ Delta Pharmaceutics Ltd., 1- 3 Manor Road, Chatham, ME4 6AE, Kent, UK

^3^University of Nicosia, School of Life and Health Sciences, Department of Pharmacy, 29th Street, No. 17, Elliniko 167 77, Athens, Greece

^4^ Medway School of Pharmacy, University of Kent, Central Avenue, Chatham Maritime, ME4 4TB, UK

* Corresponding author

*E-mail address*: [v.trivedi@kent.ac.uk](mailto:v.trivedi@kent.ac.uk) (V. Trivedi)

Table S1: Stability studies of SDs and ODTs (n=3).

| **Formulation** | **Conditions** | **FDP content at 60 min (%)** |
| --- | --- | --- |
| Day 0 | | |
| PEG 20K SD (30% w/w FDP) | - | 94.8 ± 3.5 |
| PEG 4K SD (30% w/w FDP) | - | 90.9 ± 3.1 |
| PEG 20K ODT (30% w/w FDP) | - | 94.7 ± 4.8 |
| PEG 4K ODT (30% w/w FDP) | - | 94.4 ± 3.2 |
| Day 30 | | |
| PEG 20K SD (30% w/w FDP) | Ambient | 93.6 ± 2.8 |
| PEG 4K SD (30% w/w FDP) | Ambient | 92.1 ± 2.1 |
| PEG 20K ODT (30% w/w FDP) | Ambient | 93.5 ± 3.6 |
| PEG 4K ODT (30% w/w FDP) | Ambient | 93.4 ± 3.6 |
| PEG 20K SD (30% w/w FDP) | 40 °C/75% RH | 93.8 ± 2.9 |
| PEG 4K SD (30% w/w FDP) | 40 °C/75% RH | 92.1 ± 3.1 |
| PEG 20K ODT (30% w/w FDP) | 40 °C/75% RH | 93.5 ± 3.6 |
| PEG 4K ODT (30% w/w FDP) | 40 °C/75% RH | 94.3 ± 2.6 |





Figure S1: DSC thermograms of bulk FDP, PMs, and SDs with PEG 6K





Figure S2: DSC thermograms of bulk FDP, PMs, and SDs with PEG 10K





Figure S3: DSC thermograms of bulk FDP, PMs, and SDs with PEG 20K





Figure S4: XRD diffractograms of bulk FDP, scFDP, and SDs prepared with PEG 6K





Figure S5: XRD diffractograms of bulk FDP, scFDP, and SDs prepared with PEG 10K





Figure S6: XRD diffractograms of bulk FDP, scFDP, and SDs prepared with PEG 20K





Figure S7: ATR-FTIR spectra of bulk FDP, scFDP, PMs and SDs prepared with PEG 6K





Figure S8: ATR-FTIR spectra of bulk FDP, scFDP, PMs and SDs prepared with PEG 10K





Figure S9: ATR-FTIR spectra of bulk FDP, scFDP, PMs and SDs prepared with PEG 20K
